# Comment on Torrellas et al. Effectiveness, Safety and Choroidal Changes of a Fovea-Sparing Technique for the Treatment of Chronic Central Serous Chorioretinopathy with Yellow Subthreshold Laser. *J. Clin. Med.* 2023, *12*, 1127

**DOI:** 10.3390/jcm12134302

**Published:** 2023-06-27

**Authors:** Jose Ignacio Fernandez-Vigo, Jacobo Emilio Enríquez-Fuentes, Carlos Oribio-Quinto, Antonio Domingo Alarcón-García, Francisco Javier Moreno-Morillo, Juan Donate-López

**Affiliations:** 1 Department of Ophthalmology, Hospital Clínico San Carlos, Instituto de Investigación Sanitaria (IdISSC), 28040 Madrid, Spain; 2 Centro Internacional de Oftalmología Avanzada, 28010 Madrid, Spain

Recently, we read with great interest the article by Torrellas et al. [1], in which the authors analyzed the anatomical and functional results in chronic Central Serous Chorioretinopathy after yellow subthreshold Laser (STL). We believe it to be an excellent study and the data offered could have great clinical relevance, and we would like to congratulate the authors.

However, from our point of view, some methodological and research concerns need to be clarified by the authors to support their conclusions.

First, they concluded that the best-corrected visual acuity improved in 93% of the patients and remained stable in 7%. However, in their study, the authors did not define the criteria employed regarding what they considered a visual acuity (VA) improvement or stabilization, allowing them to reach that conclusion. It is well known that VA can have slight fluctuations and variability between clinical visits, in particular when the BCVA was measured on a decimal scale and not in logMAR or ETDRS, which are more accurate.

Second, in the methods section, the authors described their procedure for the application of the laser. They outlined a power titrated to one-third of the minimum energy required to produce a minimally visible burn in the macular periphery. However, there is no information in the manuscript about the energy ultimately employed in their cases for the impacts after titration, with the mean, standard deviation, and range of energy employed, as well as the total energy delivered in an area. As pointed out by some experts in the field, the most common error and criticism made for this treatment is the lack of efficacy due to undertreatment, secondary to low energy, too few spot applications, or insufficient density and small areas treated [2,3,4].

Third, the authors stated that the spot size employed was 160 microns, delivered in a 5% duty cycle in a confluent pattern guided by OCT, AF, or FA. The treated zone was the leakage point or points on the FA and/or the areas of hyperfluorescence on the AF, as well as the whole area with SRF on OCT. However, despite the variability in regard to the patient and the amount of fluid, it would be interesting to know the data about the mean number of impacts as well as the range employed to treat the patients in this study.

Fourth, although it is clear that the main purpose was to focus on the SRF resorption, it would be interesting, if possible, to have information on the treatment response regarding a characteristic commonly present in CSC patients, namely serous pigmentary epithelium detachment (PED), and an analysis of its height or size, as well as the efficacy on patients with fibrin secondary to high hyperpermeability.

Fifth, the authors reported that the reliability of the measurements was assessed for the choroidal thickness and Haller’s, choriocapillaris, and Sattler’s layers using two independent masked retinal specialists. However, they did not define whether the SRF were performed by one or two observers and in a masked fashion in order to avoid a possible involuntary bias if the observer knows that the image corresponds to the visit after treatment.

Sixth, the authors observed that a complete resorption of the SRF was achieved in 75% of the patients with shorter disease duration (less than 12 months) after 6 months of follow-up, while 67.7% of the patients with long-standing disease had a complete response. The authors mentioned that a single STL treatment was sufficient for 41.9% of the patients. However, due to the recurrence of SRF to any degree, which was observed in 58.1% of the patients, 44.2% and 13.9% of the patients required two and three STL treatments, respectively. We believe it would be interesting if the authors could detail when the recurrences occurred in the patients treated more than once during the six-month study in order to differentiate them from the persistence of fluid. In addition, it would be interesting to know whether the authors identified some of the baseline characteristics of the patients associated with the need for retreatment and whether the latter was performed with the same parameters as the initial SLT or whether any changes were made when trying to increase the efficacy of this treatment.

As reported by Keunen et al., despite the proven effectiveness of STL, the lack of standardization has limited its clinical application and usefulness [2]. Recently, some of the authors of the present study [1], have proposed STL therapy guidelines for retinal diseases [5], which could represent a great advance. However, there is a need for a broad consensus among retina specialists as well as a need for a consensus about the criteria to assess the efficacy of the treatment, both in anatomical and functional results, in order to compare among different studies.

For all the reasons outlined, we believe that clarifications from the authors regarding the issues raised could help to improve the reproducibility of their interesting method by other researchers and improve the results of this treatment in many patients worldwide.
